# The complete chloroplast genome sequence of *Albuca bracteata* (Asparagaceae)

**DOI:** 10.1080/23802359.2026.2722775

**Published:** 2026-09-01

**Authors:** Youqing Cheng, Tianzi Wang, Yan Zhang, Jinglei Huang, Wenling Shi, Lihan Song, Guoqing Wan

**Affiliations:** ^a^ School of Pharmacy, Digital and Intelligent Empowerment Biomedical Innovation Center, Shanghai University of Medicine and Health Sciences, Shanghai, China; ^b^ School of Chemistry and Chemical Engineering, Shanghai University of Engineering Science, Shanghai, China; ^c^ Shanghai Key Laboratory of Molecular Imaging, Shanghai University of Medicine and Health Sciences, Shanghai, China

**Keywords:** *Albuca bracteata*, *Ornithogalum caudatum*, chloroplast genome, plastome, phylogenetic analysis, Asparagaceae

## Abstract

*Albuca bracteata* (syn. *Ornithogalum caudatum*) is an ornamental bulbous plant wth limited plastome data. We assembled its complete chloroplast genome (GenBank PZ425793) from high-throughput sequencing reads. The156.695-bp plastome comprised an 84,786-bp large single-copy region, an 18,555-bp small single-copy region, andtwo 26,677-bp inverted repeats, with 37.66% GC content and 533.8x mean coverage. It contained 113 unique genes79 protein-coding, 30 tRNA, and four rRNA genes). Phylogenetic analysis placed *A. bracteata* as sister to *Albuca kirki*. This plastome supports species authentication and comparative and phylogenetic studies of Ornithogaloideae.

## Introduction

*Albuca bracteata* (Thunb.) J.C.Manning & Goldblatt (2009), historically treated as *Ornithogalum caudatum* Aiton (1789), is a bulbous monocotyledon in Asparagaceae, subfamily Ornithogaloideae. The name *Albuca bracteata* is used in this manuscript to match the current NCBI Taxonomy and GenBank record for accession PZ425793; *Ornithogalum caudatum* is retained as a synonym at first mention. The species is familiar in cultivation because of its exposed green bulb, production of small bulbils, and long racemose inflorescence, and it has also appeared in folk medicinal use. Its taxonomic background is not entirely straightforward. Several species once assigned to *Ornithogalum* have been reinterpreted as molecular data accumulated, and generic boundaries within Ornithogaloideae have been revised more than once (Manning et al. 2009). In a group where vegetative and floral characters may overlap among related taxa, complete chloroplast genomes offer a comparatively stable set of characters for identification and phylogenetic assessment.

In most angiosperms, the chloroplast genome is organized into a large single-copy (LSC) region, a small single-copy (SSC) region, and two inverted repeat (IR) regions (Daniell et al. 2016). Although plastomes have long been considered relatively conserved in gene order and structure, recent large-scale analyses have revealed pervasive rearrangements that challenge this assumption (Cauz-Santos 2025); nevertheless, they still contain sufficient sequence variation for species delimitation, comparative genomics, and reconstruction of relationships among closely related taxa (Dong et al. 2018; Gitzendanner et al. 2018). In Asparagaceae specifically, complete chloroplast genomes have been successfully used for comparative and phylogenetic analyses in genera such as *Polygonatum* and *Aspidistra* (Huang et al. 2023; Yang et al. 2023). For *A. bracteata*, however, a complete and well-documented chloroplast genome has remained unavailable. We therefore assembled the complete plastome of *A. bracteata*, examined its genome structure and read coverage, and used plastome-scale data to infer its placement among representative Asparagaceae taxa. These results provide a reference for future work on germplasm identification and plastome evolution in Ornithogaloideae.

## Materials and methods

### Plant material, DNA extraction, and sequencing

Fresh leaves of *Albuca bracteata* (syn. *Ornithogalum caudatum*) were collected from Suqian, Jiangsu, China (34°09′40ʺN, 118°35′40ʺE). The plant was identified on the basis of its bulbous habit and diagnostic morphological characters (Figure 1). A voucher specimen (YXY2025007, HTWNQ) was preserved in the Herbarium of School of Pharmacy, Shanghai University of Medicine and Health Sciences. Requests for research materials should be addressed to Guoqing Wan (wangq@sumhs.edu.cn). Total genomic DNA was isolated from leaf tissue using a modified cetyltrimethylammonium bromide (CTAB) method (Doyle 1987). DNA integrity was checked by agarose gel electrophoresis and NanoDrop spectrophotometry, and DNA concentration was measured with Qubit fluorometry. A paired-end sequencing library was prepared after DNA fragmentation with a Covaris ultrasonicator, followed by end repair, A-tailing, adapter ligation, purification, and PCR amplification. Library size and concentration were evaluated using an Agilent 2100 Bioanalyzer, Qubit, and qPCR. Sequencing was carried out on the DNBSEQ-T7 platform with DNA nanoball and cPAS sequencing chemistry. The raw sequencing reads were deposited in the NCBI Sequence Read Archive under accession SRR3867003, associated with BioProject PRJNA1467247 and BioSample SAMN60212212.

**Figure 1.**
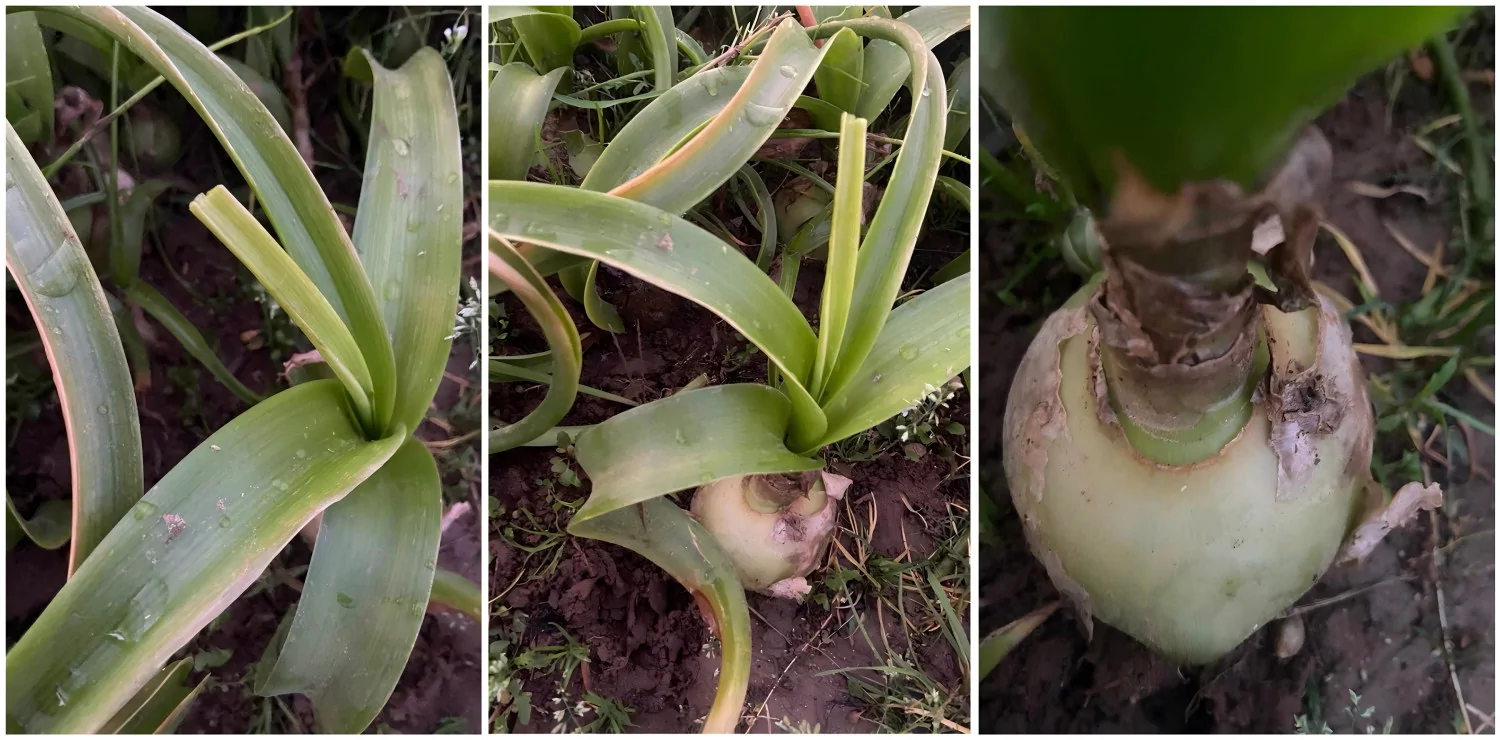
Photographs of *Albuca bracteata* (syn. *Ornithogalum caudatum*) used in this study. The specimen is characterized by a conspicuous exposed green bulb and broad, fleshy, strap-shaped leaves arranged in a basal rosette. The photographs are original images taken by the authors in Suqian, Jiangsu, China, during sample collection.

### Genome assembly and annotation

After removal of adapters and low-quality reads, the clean reads were used for chloroplast genome assembly. To reduce the complexity of organelle assembly, reads were first mapped to an in-house chloroplast genome database with Bowtie2 (Langmead and Salzberg 2012). The three chloroplast coding sequences that recruited the largest number of reads were then used as starting sequences for NOVOPlasty assembly (Dierckxsens et al. 2017). Where necessary, CAP3 was used to connect contigs, and the circular chloroplast genome was manually checked and adjusted to a consistent starting position (Huang and Madan 1999). Annotation was performed using GeSeq and tRNAscan-SE, followed by manual correction of gene boundaries (Lowe and Eddy 1997; Tillich et al. 2017). The circular map was drawn with OGDRAW (Lohse et al. 2007). To evaluate the assembly, the clean reads were mapped back to the final plastome with Bowtie2, and sequencing depth was calculated along the genome. Coverage-depth and cis-/trans-splicing gene-structure maps were prepared as Supplementary Figures S1–S3 in accordance with the journal guideline. The complete chloroplast genome was deposited in GenBank under accession PZ425793, and the corresponding raw-read record is available under SRA accession SRR3867003.

### Phylogenetic analysis

For phylogenetic analysis, complete chloroplast genome sequences of selected Asparagaceae taxa were retrieved from GenBank. The dataset comprised *Hyacinthus orientalis* (OM320803–OM320809), *Scilla siberica* (OM320810), *Bellevalia paradoxa* (OM320811), *Barnardia japonica* (KX822775, MH287351, MT319125), *Hyacinthoides non-scripta* (MN824434), *Albuca kirkii* (KX931448), *Oziroe biflora* (KX931463), and two outgroups, *Astelia pumila* (MH752984) and *Astelia australiana* (MN839533). Whole plastome sequences were aligned using Clustal Omega v1.2.4 (Sievers and Higgins 2014). A maximum-likelihood tree was inferred with FastTree v2.1.10 (Priceet al. 2010) under the generalized time-reversible (GTR) model, and branch support was estimated using the Shimodaira–Hasegawa-like local support test implemented in FastTree. Multiple accessions from the same species were labeled with their GenBank accession numbers in the tree to distinguish them from one another. Taxon selection emphasized complete plastomes from Ornithogaloideae/Scilloideae and closely related Asparagaceae lineages, while two Astelia species were used as outgroups; the analysis was designed to test the placement of the focal taxon rather than to provide exhaustive sampling across Asparagales.

## Results

The assembled chloroplast genome of *Albuca bracteata* (GenBank accession no. PZ425793) was a circular molecule of 156,695 bp, with an overall GC content of 37.66% (Table 1). Its quadripartite structure consisted of an 84,786 bp LSC region, an 18,555 bp SSC region, and a pair of IR regions of 26,677 bp each (Figure 2). Read-mapping analysis supported the assembly: the average sequencing depth across the plastome was 533.8×, and the coverage profile did not show an apparent zero-coverage gap (Supplementary Figure S1). The plastome contained 113 unique genes, comprising 79 protein-coding genes, 30 tRNA genes, and four rRNA genes. When duplicated copies in the two IR regions were counted, the annotation comprised 133 gene copies (87 protein-coding, 38 tRNA, and eight rRNA copies). No pseudogene was found. The circular map showed the expected arrangement of photosynthesis-related genes, ribosomal protein genes, RNA polymerase genes, tRNA genes, rRNA genes, and open reading frame genes across the LSC, SSC, and IR regions. The *rps12* gene showed the typical trans-splicing pattern, and several plastid genes contained introns, as commonly observed in angiosperm chloroplast genomes. The structures of cis-splicing genes and the *rps12* trans-splicing gene are shown in Supplementary Figures S2 and S3, respectively.

**Figure 2.**
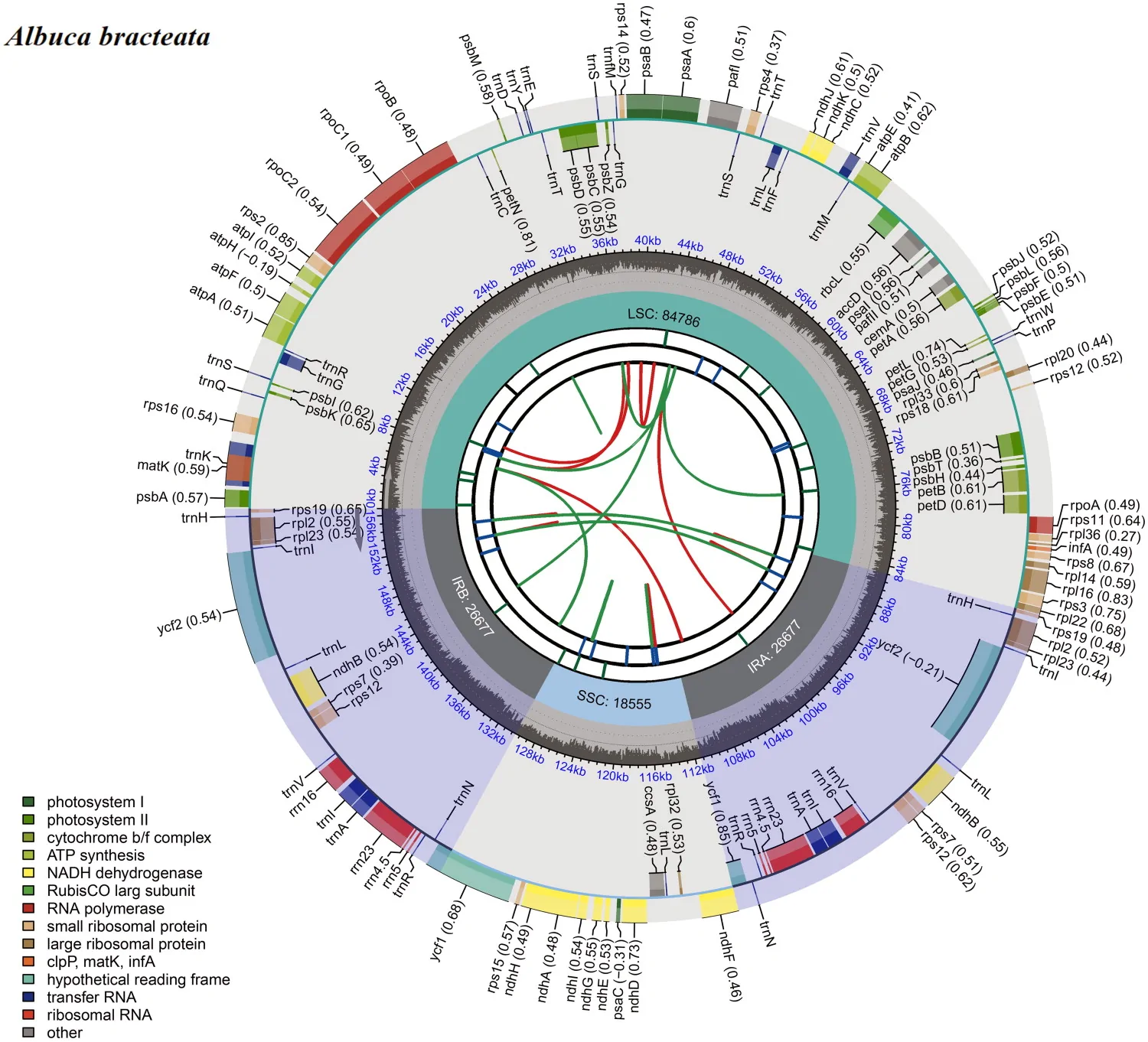
Circular chloroplast genome map of *Albuca bracteata* (syn. *Ornithogalum caudatum*) generated with OGDRAW. Genes shown outside and inside the circle are transcribed in opposite directions. The inner grey ring represents GC content. LSC, SSC, IRa, and IRb indicate the large single-copy region, small single-copy region, and two inverted repeat regions, respectively.

**Figure 3.**
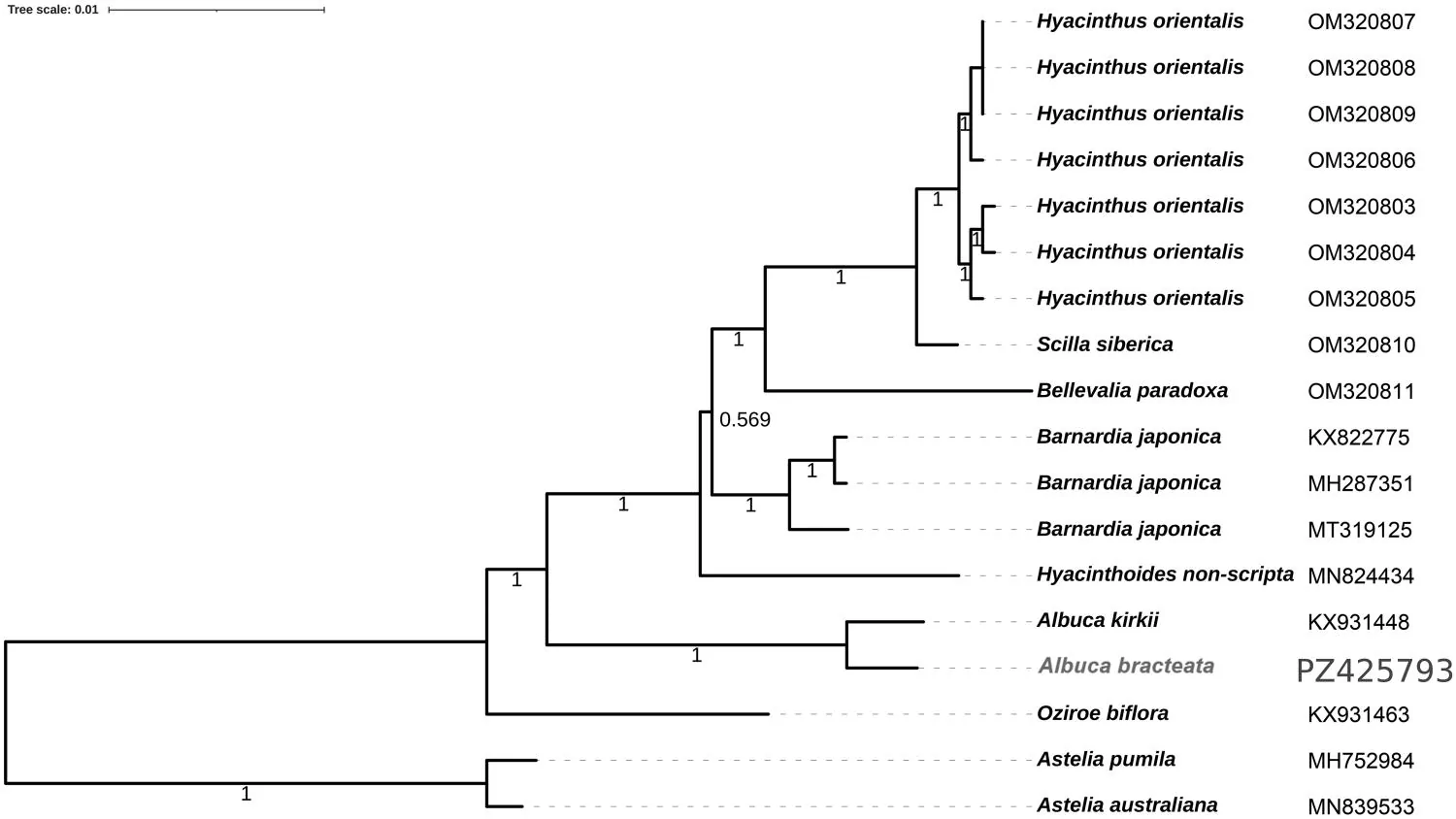
Phylogenetic tree of *Albuca bracteata* (syn. *Ornithogalum caudatum*) and related Asparagaceae taxa based on complete chloroplast genome sequences. The tree was inferred with FastTree under the GTR model, and branch support values indicate SH-like local support. Multiple accessions from the same species are distinguished by GenBank accession numbers in the tree. The following sequences were used: *Albuca bracteata* PZ425793 (this study), *Hyacinthus orientalis* OM320803-OM320809 (Wong et al. 2022), *Scilla siberica* OM320810 (Wong et al. 2022), *Bellevalia paradoxa* OM320811 (Wong et al. 2022), *Barnardia japonica* KX822775 and MH287351 (Wang et al. 2018), *Barnardia japonica* MT319125 (Chen et al. 2020), *Hyacinthoides non-scripta* MN824434 (Garnett et al. 2020), *Albuca kirkii* KX931448 and *Oziroe biflora* KX931463 (McKain et al. 2016), *Astelia pumila* MH752984 (Pfanzelt et al. 2019), and *Astelia australiana* MN839533 (Amor et al. 2020). The newly sequenced *Albuca bracteata* plastome is shown in larger type in the tree.

**Table 1.** General characteristics of the *Albuca bracteata* chloroplast genome.

Feature	Value
Total genome length	156,695 bp
Large single-copy region	84,786 bp
Small single-copy region	18,555 bp
Inverted repeat regions	26,677 bp each
Overall GC content	37.66%
Total unique genes	113
Protein-coding genes	79
tRNAs	30
rRNAs	4
Pseudogenes	0

The maximum-likelihood tree placed *A. bracteata* within Asparagaceae with strong support (Figure 3). In the recovered topology, *A. bracteata* was sister to *Albuca kirkii*, while this lineage was separated from the *Hyacinthus*, *Barnardia*, *Bellevalia*, *Scilla*, and *Hyacinthoides* lineages included in the analysis. Because complete plastome availability remains taxonomically uneven, the present topology should be interpreted as a focused test of the focal taxon’s placement rather than as a comprehensive family-level phylogeny.

## Discussion and conclusions

The plastome reported here adds a complete chloroplast genome record for *Albuca bracteata* (GenBank accession no. PZ425793). Its genome size, GC content, gene number, and LSC–SSC–IR organization fall within the range commonly seen in photosynthetic monocot plastomes. No large structural rearrangement was evident from the assembled genome. The read-mapping profile, with an average depth of 533.8× and continuous coverage across the plastome, provides additional support for the reliability of the assembly. The retention of 113 unique genes (79 protein-coding genes, 30 tRNA genes, and four rRNA genes) indicates a conserved plastid framework for photosynthesis, transcription, and translation.

The close relationship between *A. bracteata* and *Albuca kirkii* recovered in this analysis deserves attention in view of the long-standing taxonomic revisions within Ornithogaloideae. Several species historically treated under *Ornithogalum* have been transferred to other genera as molecular evidence has accumulated, and plastome data can help clarify these relationships at a broader scale. At the same time, the present inference is based on chloroplast genomes and a focused, taxonomically limited set of sampled taxa. The suggested accessions span different lineages and taxonomic levels within Asparagales, and isolated addition of distantly related taxa would not substitute for balanced sampling across Ornithogaloideae and related subfamilies. Additional sampling across *Ornithogalum*, *Albuca*, and related genera, together with nuclear markers, will be needed to test species-level relationships and to examine possible discordance between plastid and nuclear histories.

Taken together, the complete chloroplast genome of *A. bracteata* provides a reference sequence for molecular authentication of ornamental and medicinal germplasm and for comparative plastome studies in Ornithogaloideae. The genome may also support the development of chloroplast markers for population-level and phylogeographic studies of *A. bracteata* and its relatives.

## Supplementary Material

Supplemental Material

Supplemental Material

Supplemental Material

## Data Availability

The complete chloroplast genome sequence generated in this study has been deposited in GenBank under accession number PZ425793. The associated BioProject, BioSample, and SRA accession numbers are PRJNA1467247, SAMN60212212, and SRR3867003, respectively. The voucher specimen (YXY2025007, HTWNQ) and DNA materials are available from the corresponding author upon reasonable request.
